# The Relationship between Child Maltreatment and Emotion Recognition

**DOI:** 10.1371/journal.pone.0086093

**Published:** 2014-01-20

**Authors:** Michiko Koizumi, Haruto Takagishi

**Affiliations:** 1 Graduate School of Education, Hokkaido University, Sapporo, Japan; 2 Japan Society for the Promotion of Science, Tokyo, Japan; 3 Tamagawa University Brain Science Institute, Tokyo, Japan; University of Tuebingen Medical School, Germany

## Abstract

Child abuse and neglect affect the development of social cognition in children and inhibit social adjustment. The purpose of this study was to compare the ability to identify the emotional states of others between abused and non-abused children. The participants, 129 children (44 abused and 85 non-abused children), completed a children’s version of the Reading the Mind in the Eyes Test (RMET). Results showed that the mean accuracy rate on the RMET for abused children was significantly lower than the rate of the non-abused children. In addition, the accuracy rates for positive emotion items (e.g., hoping, interested, happy) were significantly lower for the abused children, but negative emotion and neutral items were not different across the groups. This study found a negative relationship between child abuse and the ability to understand others’ emotions, especially positive emotions.

## Introduction

Child abuse and neglect are critical issues worldwide. The Japanese Ministry of Health, Labor and Welfare reported 56,384 cases of child abuse and neglect in 2010; this number has been increasing annually [1]. Child abuse and neglect can be classified into four categories: physical abuse, psychological abuse, sexual abuse, and neglect [2]. Child abuse and neglect affects children physically and psychologically, as well as inhibiting social adjustment. Previous studies in Japan have shown that about 50% of juveniles who turn to delinquency were abused by their parents [3], [4], and most parents who abuse their children were also abused by their parents during childhood [5], [6]. It is well known that being abused is one of several significant risk factors for mental disorders (e.g., borderline personality disorder, mood disorder), sex crime victimization, and domestic violence, among others [7]–[9]. Furthermore, the experience of being abused affects children’s social cognition, especially the ability to understand others.

Some studies have examined the impact of abuse on the understanding of others’ emotions and beliefs [10]–[14]. Cicchetti et al. [13] reported that 3- to 8-year-old abused children had difficulty passing a false belief task, which measures the development of Theory of Mind, an ability to represent others’ beliefs [15], [16]. Pears and Fisher [11] found that abused children who lived with foster parents performed poorer on an affective perspective-taking test [17] than those who were not abused and lived with their biological parents. These two studies suggest that the family environment plays a critical role in the ability to develop an understanding of others’ beliefs and emotions. Moreover, abused children show abnormal patterns in their recognition of the facial expressions of other people. Employing the photographs of facial expressions [18], Pollak et al. [14] found that physically abused children were more likely to perceive others’ emotions as anger, while neglected children were more likely to perceive them as sad. In addition, physically abused children were sensitive to the expression of anger [10], [12]; they reacted to an angry face faster than non-abused children and had difficulty directing attention away from the angry face.

A number of studies have reported that abused children have difficulty getting along with other people [19], [20]. The reason for this may stem from a deficit in the ability to recognize the facial expressions of others and infer their beliefs and emotions from these expressions. Thus, it is important to examine the impact of being abused on the ability to understand others through facial expressions. Previous studies have employed puppet play [11] or Ekman’s photographs of facial expressions and morphing movies made from these photos [12]–[14]. However, tests using Ekman’s photographs were limited by the ease at which participants identified the emotional expressions, which may have led to a ceiling effect. Furthermore, photos of standard emotional expressions such as “angry,” “happy,” or “sad” are not suitable for measuring the ability to understand the more complex or subtle expressions seen in daily life; thus, we believe that, to identify the full impact of abuse on the ability to understand others’ emotions and infer intentions, photos with a wider variety of expressions, for example, “joking,” “bored,” and “interested,” were necessary. For these reasons, we selected the “Reading the Mind in the Eyes” Test (RMET) [20], [21] to measure more accurately the ability to understand complex or subtle expressions. The RMET was developed by research wherein people were asked to identify the emotion expressed in a photograph that showed only the eyes, and the most frequent response was considered the correct answer. Therefore, the RMET is the most suitable test to measure the level of participant's social recognition in real social situations. Our prediction was that the accuracy of abused children on the RMET would be lower than that of non-abused children.

## Materials and Methods

### Ethics Statement

This study was approved by the ethical committee at the Department of Education, Hokkaido University, Japan. The parents of the control group participants and the agent of parental authority for the abused children signed informed consent forms in advance.

### Participants

Participants were 129 Japanese children in two groups: abused and non-abused (control). There were 44 children (male = 27, female = 17) in the abused group and 85 children (male = 50, female = 35) in the control group. There was no significant difference in gender proportion between the groups (*χ* ^2^ (1) = 0.078, *p* = .78). The demographic characteristics of the two groups are shown in Table 1. The child guidance center confirmed that children in the abused group had been victims of child abuse and neglect. Children in the abused group were 6 to 17 years old (*M* = 11.7, *SD* = 2.8) and the control group children were aged 10 to 18 (*M* = 11.7, *SD* = 2.2). There was no significant difference of age between two groups (*t*(72) = 0.03, *p* = .98). We did not test for the presence of development disorder due to child welfare system regulations in Japan. The abused children lived in a child welfare institution; foster care is not common in Japan. The institution’s mission is to care for children with social adaptation or mental health problems. The children in the control group lived with their families.

**Table 1:** Participant characteristics.

	Abused Group		Control Group		*p*-value^b^
	*M*	*SD*	*M*	*SD*	
N (Male/Female)	44 (27/17)		85 (50/35)		0.78
Age	11.7	2.8	11.7	2.2	0.98
AQ ^a^ total	22.5	7.9	15.1	6.2	<.0001

Notes: ^a^AQ: Autism Quotient, Japanese version;

^b^χ^2^ test for gender, t-tests for all others.

In the public child welfare system in Japan, specific categories of maltreatment (e.g., physical abuse, psychological abuse, sexual abuse, and neglect) experienced by a child are protected, along with the identity of the abuser, socioeconomic status, and home environment, so we could not access this information even though our purpose was academic and the children’s identities would be protected. Therefore, it was not possible to examine the impact of these variables on RMET performance without the assistance of the child welfare institution.

### Reading the Mind in the Eyes Test

All participants completed the child version of the RMET [20]. This version of the RMET has 28 items and measures the ability to understand the emotional states of others. Participants are asked to look at pictures of human eyes and to choose the emotion that best fits the picture from four possibilities. Previous studies have found that adults with Asperger syndrome or high functional autism were less accurate than adult participants without autism spectrum disorder [21]. In the child version of the RMET, adults read the four choices aloud and children circled the answer they chose.

### Autism Spectrum Quotient (AQ)

The AQ measures the symptoms of autism spectrum disorder. This scale has 50 items and is divided into 5 subscales: social skills, attention switching, attention to detail, communication, and imagination. Higher scores are associated with more autism-spectrum symptoms. The adult version of this test is a self-reported questionnaire; for the child version, the child's guardian reads the questions and the child responds. In this study, we used the Japanese version of the AQ [23]. This test is a low stress situation for the child, which is particularly important when working with abused children, because it is administered by a parent or guardian. As the child participants in this study may have a developmental disorder, we used the child version of the AQ to control for the effect of Pervasive Development Disorder (PDD) on RMET performance.

### Data Analysis

In line with the previous studies using the RMET, the 28 items were classified by their emotional valence: positive (7 items), negative (10 items), or neutral (11 items) [21]. The classification of these items is summarized in Table 2. Because the number of items in each valence was different, we used the accuracy rate in the analyses.

**Table 2:** Classification of RMET items by emotional valence.

Positive (7 items)		Negative (10 items)		Neutral (11 items)	
Item No.		Item No.		Item No.	
1	Kind	2	Sad	5	Making somebody do something
3	Friendly	4	Upset	8	Remembering
7, 19, 21	Interested	6, 25	Worried	9, 13, 14, 22	Thinking about something
11	Hoping	10, 15, 27	Not believing	12, 24	Serious
28	Happy	17	A bit worried	16	Made up her mind
		18	Thinking about something sad	23	Sure about something
		20	Not pleased	26	Nervous

First, we compared the mean level of AQ score between the abused and non-abused groups. Second, we compared the mean accuracy rate for each emotion valence (positive, negative, and neutral) between the abused and non-abused groups. Finally, using a multiple regression analysis, controlling for age, gender, and the AQ score, we tested the effect of being abused on emotion recognition.

## Results

**Figure 1:**
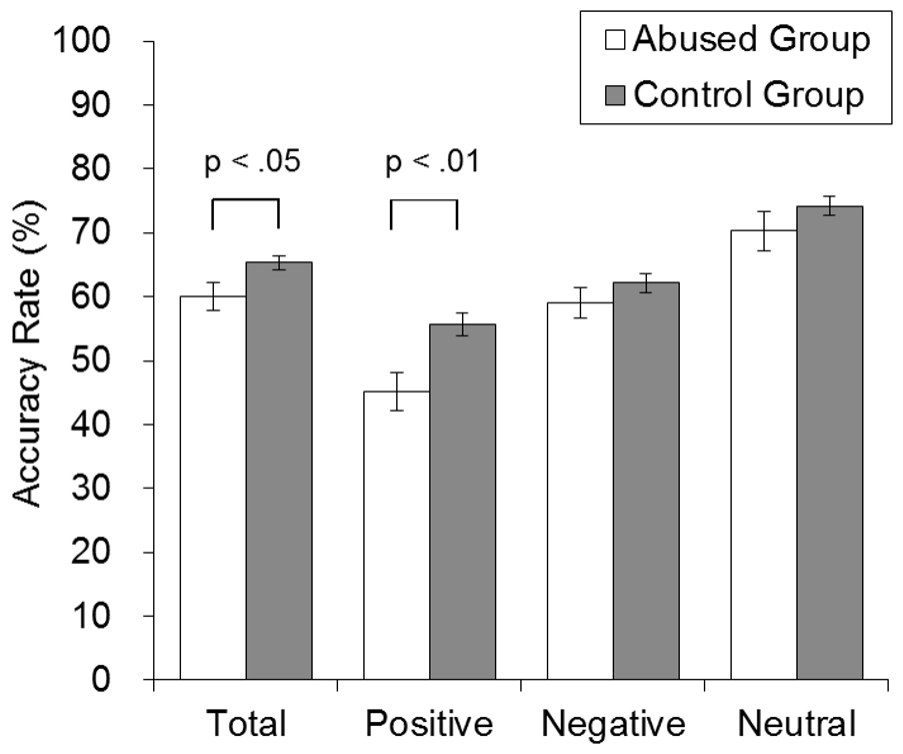
Mean accuracy rate for each emotional valence by group. Error bars represent standard errors.

The mean AQ score of the abused group was significantly higher than that of the control group (abused group: *M* = 22.5, *SD* = 7.9; control: *M* = 15.1, *SD* = 6.2; *t*(70) = 5.3, *p*<.001, *d* = 1.08) (Table 1). The performance scores on the RMET are shown in Figure 1. The mean accuracy rate on the RMET of the abused group was significantly lower than was the control group accuracy (abused group: *M* = 0.60, *SD* = 0.15; control: *M* = 0.65, *SD* = 0.09; *t*(60) = 2.19, *p* = .032, *d* = 0.44). The score for positive emotional valence in the abused group was lower than that in the control group (*t*(127) = 3.12, *p* = .002, *d* = 0.61), but the difference in scores for negative and neutral emotional valences was not significant (negative: *t*(127) = 1.20, *p* = .23, *d* = 0.21, neutral: *t*(63) = 1.16, *p* = .25, *d* = 0.24).

Finally, a regression analysis revealed that there was a significant positive effect of age (*β* = .23, *p* = .008) and negative effect of group (control = 0; abused group = 1) (*β* = −.22, *p* = .022) on position emotion RMET score, but the effects of gender (female = 0, male = 1) (*β* = .07, *p* = .41) and AQ score (*β* = −.08, *p* = .39) were not significant.

## Discussion

Our findings revealed that the accuracy of the abused children on the RMET was significant lower than the accuracy of the control group. Interestingly, this pattern was observed in only the identification of positive emotions. Furthermore, the impact of being abused on the ability to recognize positive emotion remained low after the effects of age, gender, and AQ score were removed. These results indicated that abused children were less able to recognize positive emotional expressions. Why did abused children have difficulty inferring positive emotions from facial expressions? One explanation may be that the abused children had less exposure to positive emotions from their parents than did non-abused children and may have seen their parents’ negative emotional expression more often, perhaps even excessively. Therefore, abused children could identify negative expressions as well as non-abused children, but they could not identify positive expression. Indeed, Pollak et al. [14] suggested that fewer learning opportunities might affect a neglected child’s ability to discern or discriminate others’ emotions. According to embodied cognition theory [22], people understand others’ emotion using their own sensorimotor experiences. Therefore, poor emotion recognition in abused children may stem from less experience with positive emotions.

A second possible explanation is based on the inconsistency between a parent’s facial expression and future outcomes, such as the abuse that a child is subject to in an abusive family. In interpersonal situations, adults and children anticipate future outcomes from the cues found in others’ facial expressions. If others look like happy, positive outcomes are anticipated. If others look angry, negative outcomes are anticipated. According to a classic psychological experiment, infants estimate their own safety from a parent’s facial expression [23]. Negative facial expressions, such as angry or sad, from their parents make children upset, and positive facial expressions, such as joy and happiness, put children at ease. However, parents’ positive facial expressions are not always a sign of a positive future outcome in abusive families. As abusive parents sometimes harm their children while smiling, children may not associate a positive expression with a positive outcome, and they may have trouble learning to recognize positive expressions as a result. On the other hand, a strong association between negative expressions and violence has been observed in abusive families, so children may become more sensitive to negative expressions to protect themselves. Previous studies have shown that physically abused children were sensitive to angry facial expressions [24]. Thus, the deficits in social cognition observed in abused children may be the result of adaptations engendered by living in an abusive family.

A third possible explanation comes from cognitive neuroscience. Using MRIs, recent research has demonstrated that the experience of being abused affects some areas of a child’s brain [25], [26]. Tomoda and colleagues [25] showed that the experience of witnessing domestic violence reduced children’s gray matter volume and the thickness of the visual cortex. Other studies have found a reduction of gray matter volume in the hippocampus in adults participants who were abused by their parents in childhood [27], and in the medial orbitofrontal cortex and middle temporal cortex in abused children [28]. As the medial orbitofrontal cortex is the area of the brain that processes emotion recognition [29], [30], and a recent functional MRI study demonstrated that the orbitofrontal cortex area is activated when participants identified positive emotions on the RMET [31], the RMET performance of abused children might be affected by a deficit in the medial orbitofrontal cortex.

There are some limitations to the present study. Unfortunately, we could not obtain permission to use restricted information about the abused children (e.g., the type of child abuse, the age at which abuse occurred, the identity of the abuser, and treatment information). In Japan, it is difficult to obtain such information for use in academic research. As previous research has shown differential impacts of the type of child maltreatment on a child’s cognition and brain [26], [27], we need to examine the impact of the type of abuse and the child’s age when abused on the ability of understanding the emotions of others in Japanese children.

## Conclusions

Further research is needed to better understand the impact of child abuse on social cognition; we should use fMRIs to examine the relationship between the reduction of gray matter volume in child’s brain or dysfunction in the area of social brain (e.g., amygdala, medial prefrontal cortex, superior temporal sulcus, and other areas) and the performance on emotional recognition tasks. Longitudinal research, which could conduct fMRIs and emotion recognition tasks in the same samples repeatedly, could assess the impact of abuse on children properly. Thus, we could address the question of whether a child’s brain was damaged by being abused. By doing so, more specific therapeutic interventions can be developed to improve abused children’s interpersonal communications.
